# Altered activity of the medial prefrontal cortex and amygdala during acquisition and extinction of an active avoidance task

**DOI:** 10.3389/fnbeh.2015.00249

**Published:** 2015-09-15

**Authors:** Xilu Jiao, Kevin D. Beck, Catherine E. Myers, Richard J. Servatius, Kevin C. H. Pang

**Affiliations:** ^1^Neurobehavioral Laboratory, Veterans Bio-Medical Research Institute (VBRI)East Orange, NJ, USA; ^2^Neurobehavioral Research Laboratory, Department of Veterans Affairs, New Jersey Health Care SystemEast Orange, NJ, USA; ^3^Department of Pharmacology, Physiology and Neuroscience, Rutgers Biomedical Health SciencesNewark, NJ, USA; ^4^Syracuse VA Medical Center, Department of Veterans AffairsSyracuse, NY, USA

**Keywords:** c-Fos, gamma-aminobutyric-acid (GABA), intercalated cell (ITC), glutamic acid decarboxylase (GAD), parvalbumin, lever-press, rat

## Abstract

Altered medial prefrontal cortex (mPFC) and amygdala function is associated with anxiety-related disorders. While the mPFC-amygdala pathway has a clear role in fear conditioning, these structures are also involved in active avoidance. Given that avoidance perseveration represents a core symptom of anxiety disorders, the neural substrate of avoidance, especially its extinction, requires better understanding. The present study was designed to investigate the activity, particularly, inhibitory neuronal activity in mPFC and amygdala during acquisition and extinction of lever-press avoidance in rats. Neural activity was examined in the mPFC, intercalated cell clusters (ITCs) lateral (LA), basal (BA) and central (CeA) amygdala, at various time points during acquisition and extinction, using induction of the immediate early gene product, c-Fos. Neural activity was greater in the mPFC, LA, BA, and ITC during the extinction phase as compared to the acquisition phase. In contrast, the CeA was the only region that was more activated during acquisition than during extinction. Our results indicate inhibitory neurons are more activated during late phase of acquisition and extinction in the mPFC and LA, suggesting the dynamic involvement of inhibitory circuits in the development and extinction of avoidance response. Together, these data start to identify the key brain regions important in active avoidance behavior, areas that could be associated with avoidance perseveration in anxiety disorders.

## Introduction

Avoidance is a common feature of anxiety disorders (American Psychiatric Association, 2000). As avoidance behavior is a key behavioral component of anxiety disorders, learning to extinguish such behavior is a fundamental concept embedded in cognitive behavioral therapy for anxiety disorders, including post-traumatic stress disorders (PTSD) and phobias (Rau et al., 2005; Rauch et al., 2006). Thus, a better understanding of the neurobiological basis of active avoidance and its extinction will provide important insights into future behavioral and pharmacological treatment for clinical anxiety.

Malfunctions in medial prefrontal cortex (mPFC)—amygdala circuit have been identified in patients suffering PTSD, social anxiety disorder (SAD) and general anxiety disorder (GAD; Schwartz and Rauch, 2004; Cottraux, 2005; Guyer et al., 2008). Imaging studies indicate that one of the most consistent findings in PTSD patients is *hypoactive* ventral mPFC combined with *hyperactive* amygdala following provocation (Milad et al., 2006; Phan et al., 2006; Rauch et al., 2006). Avoidance develops slowly over time in anxiety disorders, so avoidance learning in animals may provide an opportunity to study the dynamic and progressive neurobiological changes associated with the development of anxiety disorders.

In animal studies, brain regions associated with avoidance behavior include prefrontal cortex (PFC), and amygdala, as well as hippocampus, striatum, medial septum and periaquaductal gray (Kirkby and Kimble, 1968; Bailey et al., 1986; Quirk and Gehlert, 2003; Mobbs et al., 2007; Straube et al., 2009; Pang et al., 2011; Cominski et al., 2014). Electrolytic lesion of the infralimbic cortex (IL) impaired active avoidance learning but facilitated freezing behavior in rats, while central amygdala (CeA) lesion resulted in the opposite behavioral changes (Moscarello and LeDoux, 2013). Rats that previously failed to learn shuttle avoidance can acquire such task following CeA lesion, suggesting ventral mPFC and CeA are playing opposite roles in avoidance learning (Choi et al., 2010). Using an Immunocytochemistry (ICC) approach, Duncan et al. reported that shuttle-box avoidance elicited c-Fos activity in the mPFC, cingulate cortex (CG), and medial amygdala in rats (1996). We recently showed that elevated c-Fos activation in mPFC is associated with faster extinction in rats (Jiao et al., 2011). Elevated and prolonged neural activity in mPFC was also observed in well-trained SD rats, represented by delta-FosB accumulation using Western blot (Perrotti et al., 2013). In addition, we found that rats that exhibited deficits in avoidance extinction also displayed lower gamma-aminobutyric acid (GABA) neuron counts and neuronal activation in basolateral amygdala, suggesting inhibitory modulation is important to ensure successful extinction (Jiao et al., 2011). The present study was conducted to further define the activity of inhibitory neurons in the mPFC and amygdala during the acquisition and extinction of lever-press avoidance.

## Materials and Methods

### Animals

Sixty-six male Sprague-Dawley (SD) rats (approximately 60 days of age at the start of the experiment) were obtained from Harlan Laboratories (Indianapolis, IN) and housed in individual cages with free access to food and water. Rats were housed in a room maintained on a 12:12 h light/dark cycle for at least 2 weeks prior to the start of the experiment. Experiments occurred between 0700 and 1700 h in the light portion of the cycle (light onset occurred at 0600 h). All procedures received prior approval by the VA NJ Health Care System Institutional Animal Care and Use Committee in accordance with AAALAC standards.

### Lever-Press Avoidance Training

As previously described (Servatius et al., 2008), training was conducted in 16 identical operant chambers (Coulbourn Instruments, Langhorn, PA) enclosed in 16 sound-attenuated boxes. The unconditional stimulus (US) was a scrambled 1.0-mA electric foot-shock delivered through the grid floor (Coulbourn Instruments, Langhorn, PA). The CS was a 1000-Hz 75-dB tone (10 dB above background noise). The inter-trial interval (ITI) was 3 min in duration and signaled by a blinking light above the lever.

The avoidance training procedure was composed of 10 sessions of acquisition (A01–A10) and six sessions of extinction (E01–E06), based on previous studies (Servatius et al., 2008; Beck et al., 2010). Avoidance training consisted of 20 trials per session. A session occurred three times per week (sessions separated by 2–3 days). Each session began with a 60 s stimulus-free period. A trial commenced with the delivery of the auditory CS. During the acquisition phase, a lever-press during the first 60 s shock free (warning) period turned off the CS and prevented the delivery of US; this response was designated an “avoidance” response. If no avoidance response was made, a shock period (shock duration = 0.5 s, inter-shock interval = 3 s, 100 shocks maximum/trial) was initiated 60 s after the start of the trial. The CS was presented during the warning and shock periods. Following a lever press during the shock period or if the maximum shock period elapsed, the CS and shock co-terminated and the ITI was initiated. A lever press during the shock period was designated an “escape” response. Extinction sessions were similar to acquisition sessions except no shocks were delivered during trials. “Avoidance” responses during the extinction sessions were lever presses with latencies less than 60 s. A rat that failed to emit a lever press response by the end of the fourth acquisition session was removed from the study. Six rats were dropped from the study for this reason.

Neural activity was assessed at four times in acquisition and two times in extinction. Rats were randomly assigned to be sacrificed after the 2nd, 4th, 8th or 10th acquisition session (session A02, A04, A08 or A10) or after the 1st or 6th extinction session (session E01 or E06) based on A% data with stratification after session A01, and are referred to as group ACQ02, ACQ04, ACQ08, ACQ10, EXT01 and EXT06, respectively.

Data analysis. One-way ANOVA design with main factor of group was used to study the dependent measures in each session to determine whether differences occurred between groups on avoidance ratio, escape ratio, and shock number during the acquisition phase, and avoidance ratio during the extinction phase. A second ANOVA with repeated measurement of session was conducted for each group to assess the change in behavior across acquisition and extinction sessions. *Post hoc* testing was conducted using Tukey’s test for pair-wise comparison between groups. All data are expressed as means ± the standard error of the mean. Due to recording errors, data from two rats in group EXT01 and 1 rat in group EXT06 were missing from session A01. Therefore, the missing subjects were not included in the analysis for session A01.

### Immunocytochemistry (ICC)

Ninety–120 min after the end of a session (A02, A04, A08, A10, E01 or E06), rats were deeply anesthetized with sodium pentobarbital (150 mg/kg) and transcardially perfused with 200 ml of saline solution, followed by 200 ml of 4% paraformaldehyde solution. Brains were extracted, post-fixed in 4% paraformaldehyde at 4°C overnight, and then stored at 4°C in 0.1 M phosphate buffer (PB) solution containing 30% (w/v) sucrose until the brains sank.

Coronal brain sections (50 microns) were prepared on a freezing microtome, and every 6th sections collected from the mPFC (bregma: +4.20 mm ~ +2.53 mm) and the amygdala (bregma: −2.04 mm ~ −3.24 mm; Paxinos and Watson, 1998) were immunostained. To reveal the neural activity during acquisition and extinction of avoidance learning, we quantified c-Fos immunoreactivity (ir; a product from the expression of the immediate early gene c-fos) as a marker of neural activity (Chaudhuri et al., 2000). Given the important role of inhibitory circuits especially in anxiety (McCabe et al., 2004; Berretta et al., 2005), we were particularly interested in the activation of inhibitory (mostly GABAergic) neurons. We stained for parvalbumin (PV), as previously described, to detect GABAergic neurons (Jiao et al., 2011). PV, a calcium-binding protein, is expressed in more than 55% of GABAergic neurons in the basal amygdala (BA) in various species (Sorvari et al., 1996; Ambalavanar et al., 1999; Kemppainen and Pitkänen, 2000; Gabbott et al., 2006; Dávila et al., 2008), and in about 35% mPFC GABAergic neurons in rat (Gabbott and Bacon, 1996; Gabbott et al., 2006). However, some PV-ir negative neurons could be GABAergic neurons. In the intercalated cell clusters (ITCs), which is composed of groups of small to medium size fast-firing GABAergic neurons located between BA and CeA (Royer et al., 1999; Royer and Paré, 2002; Mańko et al., 2011), anti-glutamic acid decarboxylase isoform 67 (GAD67, the rate-limiting enzyme of GABA synthesis) antibody was used to define GABAergic neurons (Izumi et al., 2011). Double labeling of c-Fos and PV or GAD67-ir was assessed to evaluate selective neuronal activation in each region of interest (ROI).

ICC procedures were conducted as previously described (Pang et al., 2001; Miller et al., 2005; Jiao et al., 2011). Sections were stained for c-Fos, followed by a second staining for PV or GAD67. Briefly, sections were incubated in rabbit anti-c-Fos IgG (sc-52, 1:1000, Santa Cruz, CA), mouse anti-PV (P3088, 1:1000, Sigma-Aldrich, MO), or mouse anti-GAD67 (MAB5406, 1:1000, Chemicon, CA); sections for c-Fos and PV staining were incubated overnight at room temperature while sections for GAD67 staining were incubated for 48 h at 4°C. Following incubation in primary antibodies, sections were incubated in secondary antibodies (biotinylated donkey anti-rabbit IgG, or biotinylated donkey anti-mouse IgG (1:200, Jackson ImmunoResearch, PA) for 2 h at room temperature. Visualization was performed using the avidin-biotin method (Vector Laboratories, Burlington, CA) with nickel-enhanced diaminobenzidine for c-Fos and diaminobenzidine alone for PV, or GAD67.

c-Fos-ir nuclei were counted in all ROIs; double labeled c-fos/PV-ir perikarya were counted in the anterior CG, prelimbic (PL), and IL cortices of the mPFC, and the lateral amygdala (LA)/BA; only c-Fos nuclei were counted in the ITCs (defined by darker GAD-ir area). Estimates of the number of immunostained neurons or nuclei were obtained using standard stereology procedures (West, 1993; West et al., 2009) and were conducted blind to the training conditions of the animal. Volume measures for each of the brain regions were also determined. The optical fractionator method (Stereo Investigator v.7.0, MicroBrightField, Colchester, VT) was used to obtain the estimates of cell number on a microscope with an *x-*, *y-*, *z*-axis motorized stage (ASI MS-2000, Applied Scientific Instrumentation, Eugene, OR). Cells containing c-Fos- and c-fos/ PV-ir double labeling were identified using a 40× objective lens. Double-labeled cells were defined by observing a PV-positive soma (light brown in cytoplasm) with a black nucleus in the center (c-Fos). The counting frame had a height of 10 μm and was 80 μm × 80 μm in size for basal and LA, 150 um × 100 um in size for CeA, 50 um × 100 um in size for ITC, and 50 μm × 50 μm in size for medial prefrontal area. Seven to 8 animals per group were counted for analysis in mPFC regions, 5–7 animals per group were counted in BA, and 5–6 animals per group were counted in the CeA and ITC.

Data analysis. A one-way ANOVA with main factor of group (sacrifice time) was used to assess for differences in neural activity within each ROI. General neural activity was represented by the density of c-Fos-ir cells (number of c-Fos-ir cells/volume) while GABAergic activation was represented by the ratio of the density of c-Fos—PV-ir double labeled neurons to the density of single labeled PV-ir neurons. Neural activation was also compared between phases (acquisition or extinction). *Post hoc* testing was conducted using Tukey’s test for pair-wise comparison between groups. Additional *t*-tests were performed to compare c-Fos-ir cells and activation in PV-ir neurons between ACQ10 and EXT01 or EXT6. All data are expressed as means ± the standard error of the mean.

## Results

### Acquisition and Extinction of Lever-Press Avoidance

**Figure 1:**
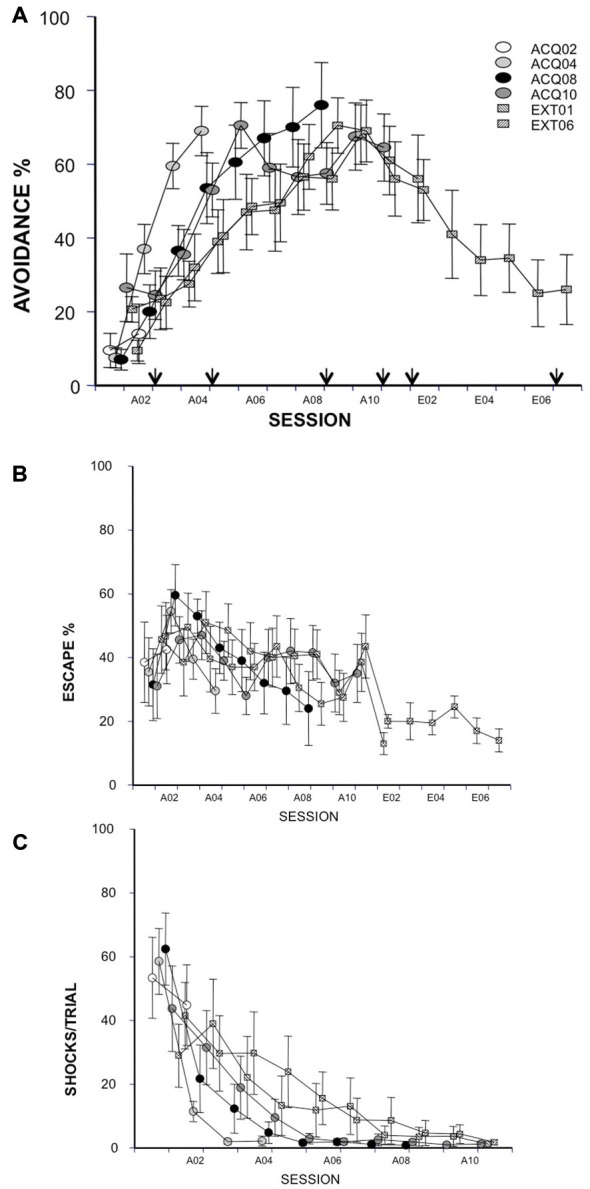
**Avoidance response, escape response and shock number received in acquisition (10 sessions) and extinction (6 sessions) were expressed as avoidance (A, arrows indicate time points of c-Fos-ir evaluation) and escape (B) ratios and the average numbers of received shocks in each session during acquisition (C)**. Each data point represents group mean ± S.E.M. (*n* = 8–10/group; data of 2 subjects from group EXT01 and 1 subject from group EXT06 were lost from session A01 due to a power failure during training).

As judged by avoidance ratio, all groups acquired the task similarly at sacrifice time (main factor = group, *ps* > 0.05). Groups EXT01 and EXT06 extinguished similarly in the first extinction session, *p* > 0.05 (Figure 1A). No group differences were found for any of the other measures (i.e., escape ratio and shock number per session, Figures 1B,C; *ps* > 0.05). As expected, rats avoided more in later acquisition sessions compared to early sessions in all groups (ACQ04, *F*_(3,27)_ = 33.48; ACQ08, *F*_(7,63)_ = 20.23; ACQ10, *F*_(9,81)_ = 5.43; EXT01, *F*_(8,72)_ = 5.66; EXT06, *F*_(8,72)_ = 6.15; *ps* < 0.0001) except for group ACQ02. Moreover, the number of shocks reduced with training (ACQ04, *F*_(3,27)_ = 30.69; ACQ08, *F*_(7,63)_ = 16.08; ACQ10, *F*_(9,81)_ = 6.1; EXT01, *F*_(8,72)_ = 3.04; EXT06, *F*_(8,72)_ = 3.18; *ps* < 0.01). During extinction, rats avoided less in later extinction sessions compared to early extinction sessions (EXT06, *F*_(5,45)_ = 3.89, *p* < 0.01). These data suggest that rats in each of the groups acquired and extinguished avoidance responses similarly and that observed difference in immediate early gene product is likely resulting from training phases (i.e., acquisition vs. extinction) and training stages (i.e., session).

### Neural Activity of the mPFC

#### Acquisition

**Figure 2:**
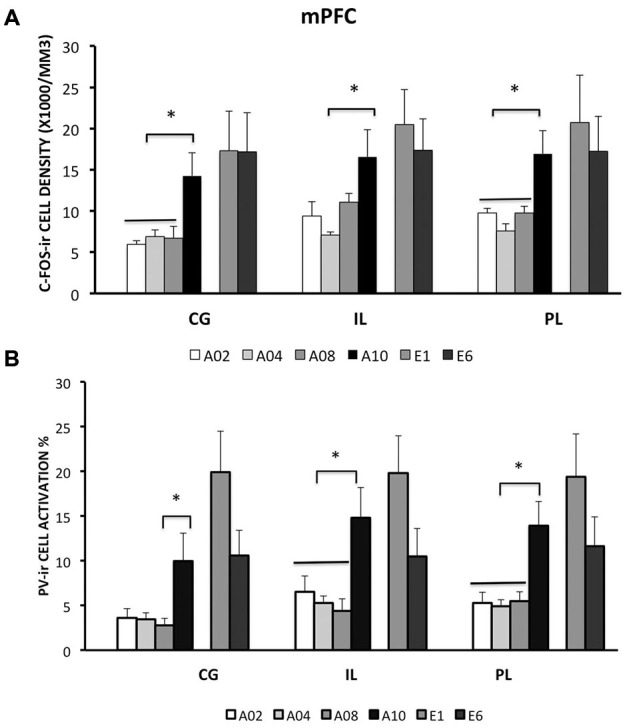
**Densities of c-Fos-ir cells and the percent of activated PV-ir cells in the medial prefrontal cortex (mPFC) (cingulate cortex, Cg; prelimbic cortex, PL; and infralimbic cortex, IL), were depicted**. **(A)** Densities of c-Fos-ir cells in mPFC sub-regions. **(B)** Activation of PV-ir cells in the mPFC. Each data point represents group mean ± S.E.M. (*ps < 0.05; *n* = 7–8/group).

Rats from ACQ10 exhibited the highest number of c-Fos-ir cells compared to other acquisition groups (Figure 2A), suggesting that mPFC neurons are still active during asymptotic avoidance performance, (CG: *F*_(3,25)_ = 5.78; IL: 4.69; PL: 7.29; *ps* < 0.01), *post hoc* *ps* < 0.05. Importantly, a greater activation of PV-ir neurons of the CG, PL and IL was observed also in ACQ10 compared to earlier acquisition sessions (CG: *F*_(3,25)_ = 3.87, *p* < 0.05; PL: *F*_(3,25)_ = 5.46, *p* < 0.005; IL: *F*_(3,25)_ = 7.75, *p* < 0.001), *post hoc* *ps* < 0.05, suggesting enhanced inhibitory tone in the mPFC as active avoidance response is fully developed (Figure 2B). (for detailed analysis results, see Table 1).

**Table 1:** **Statistical report of densities of c-Fos-ir and percent of activated PV-ir cells in ROIs during acquisition or extinction phase in rats**.

ROI	Factors	df()	*p*-value [cFos-ir]	*F*-value	*p*-value [PV-ir]	*F*-value	*p*-value [PV/cFos-ir]/[PV-ir]%	*F*-value
CG	PHASE (A vs. E)	1.42	*p = 0.002061	10.79	*p* = 0.375824	0.8	*p = 0.000109	18.25
	GROUP:A	3.25	*p = 0.003798	5.78	*p* = 0.172296	1.8	*p = 0.021156	3.87
	GROUP:E	1.13	*p* = 0.985742	0	*p* = 0.457561	0.59	*p* = 0.116396	2.83
IL	PHASE (A vs. E)	1.42	*p = 0.002553	10.30	*p* = 0.706402	0.14	*p = 0.00523	8.68
	GROUP:A	3.25	*p = 0.009889	4.69	*p* = 0.341157	1.17	*p = 0.004285	5.64
	GROUP:E	1.13	*p* = 0.596609	0.29	*p* = 0.989788	0	*p* = 0.102325	3.09
PL	PHASE (A vs. E)	1.42	*p = 0.006186	8.31	*p* = 0.91058	0.01	*p = 0.002226	10.61
	GROUP:A	3.25	*p = 0.001134	7.29	*p = 0.006574	5.15	*p = 0.000796	7.75
	GROUP:E	1.13	*p* = 0.63967	0.23	*p* = 0.818013	0.06	*p* = 0.217036	1.68
BA	PHASE (A vs. E)	1.34	*p = 0.001534	11.87	*p = 0.002308	10.86	*p = 0.001681	11.64
	GROUP:A	3.21	*p* = 0.084539	2.53	*p* = 0.101036	2.35	*p = 0.017232	4.24
	GROUP:E	1.9	*p* = 0.372144	0.88	*p* = 0.757981	0.10	*p* = 0.585622	0.32
LA	PHASE (A vs. E)	1.34	*p = 0.010545	7.33	*p* = 0.276316	1.22	*p = 0.001673	11.65
	GROUP:A	3.21	*p* = 0.124649	2.15	*p* = 0.806754	0.33	*p* = 0.610954	0.62
	GROUP:E	1.9	*p* = 0.92212	0.01	*p* = 0.561943	0.36	*p* = 0.717509	0.14
			**[cFos-ir]**
CE	PHASE (A vs. E)	1.32	*p = 0.001938	11.4
	GROUP (A)	3.18	*p = 0.004185	6.28
	GROUP (E)	1.10	*p* = 0.620524	0.26
			**[cFos-ir]**
lITC	PHASE (A vs. E)	1.33	*p = 0.001611	11.81
	GROUP (A)	3.19	*p = 0.045495	3.23
	GROUP (E)	1.10	*p* = 0.8436	0.04
mITC	PHASE (A vs. E)	1.32	*p = 0.005616	8.82
	GROUP (A)	3.18	*p = 0.002965	6.78
	GROUP (E)	1.10	*p* = 0.7765	0.09

*Denotes ps < 0.05.

#### Extinction

Compared to the acquisition phase, all three sub-regions of the mPFC had greater neural activity during the extinction phase than acquisition phase (CG: *F*_(1,42)_ = 10.79; IL: 10.3; PL: 8.31; *ps* < 0.01; Figure 2A). However, c-Fos-ir did not differ between ACQ10 and EXT01 nor between EXT01 and EXT06, suggesting *enhanced* mPFC activity might be the continuation of mPFC activity in late acquisition while c-Fos-ir cell counts sustained during extinction when response dropped. In addition, a greater proportion of PV-ir neurons was activated in all three sub-regions of mPFC during extinction compared to acquisition phase (CG: *F*_(1,42)_ = 18.25; IL: 8.68; PL: 10.61; *ps* < 0.01; Figure 2B). Interestingly, there is a trend showing PV-ir neurons are more activated in EXT01 compared to, ACQ10 in CG (*t*_(13)_ = 1.77, *p* = 0.10), and EXT06 in CG and IL (*t*_(13)_ = 1.68 and 1.76, *ps* = 0.11 and 0.10), suggesting greater inhibitory activity is associated with the transition to extinction.

## Neural Activity in Sub-Nuclei of the Amygdala

### BA and LA

#### Acquisition

**Figure 3:**
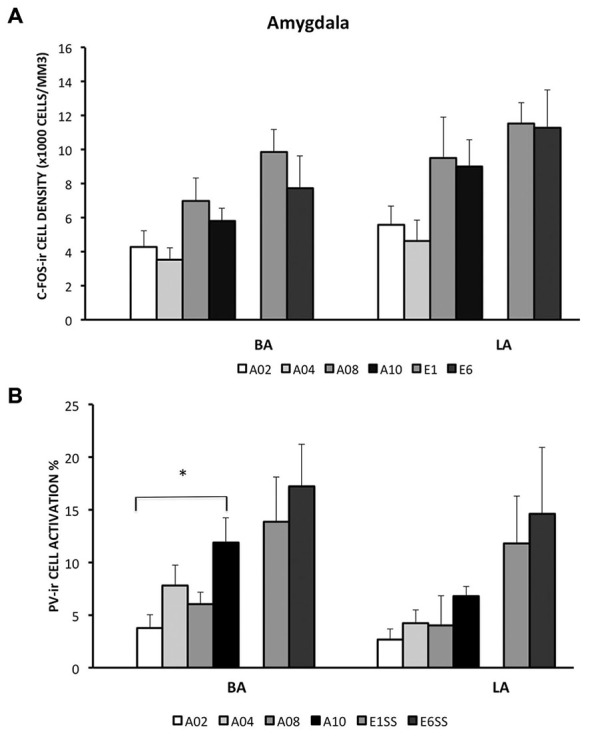
**Densities of c-Fos-ir cells and the percent of activated PV-ir cells in the LA and BA were depicted**. **(A)** Densities of c-Fos-ir cells in the lateral amygdala (LA) and basal amygdala (BA). **(B)** Activation of PV-ir cells in the LA and BA. Each data point represents group mean ± S.E.M. (*p < 0.05, *n* = 5–7/group).

In the LA and BA nuclei, the numbers of c-Fos-ir cells remained the same during acquisition sessions (*ps* > 0.05; for detailed analysis results, see Table 1). However, activity of inhibitory PV-ir neurons in the BA increased as acquisition proceeded (*F*_(3,25)_ = 4.24, *p* = 0.0172), while activity of inhibitory PV-ir neurons in the LA did not change across acquisition sessions, *p* > 0.05. The increased activity of inhibitory neurons in the BA as avoidance is acquired may be due to increased glutamatergic inputs from mPFC. Post hoc analysis showed greater BA PV-ir activation in ACQ10 compared to ACQ02 group, *p* < 0.05 (Figure 3B).

#### Extinction

Compared to the acquisition phase, BA and LA were activated to a greater extent during the extinction phase (*F*_(1,34)_ = 11.87 (BA) and 7.33 (LA), *ps* < 0.005 and 0.05 respectively), suggesting *enhanced* BA activity during extinction learning (Figure 3A). Particularly in the BA, there were more c-Fos-ir cells from EXT01 compared to ACQ10, *t*_(11,2.76)_, *p* < 0.05, suggesting increased BA activity is associated with transition to extinction. Greater activity of inhibitory PV-ir neurons was observed in both the BA and LA during extinction compared to the acquisition phase, *F*_(1,34)_ = 11.64 (BA) and 11.65 (LA), *ps* < 0.005 (Figure 3B). However, neither c-Fos-ir nor activated PV-ir neurons, altered between early and late extinction sessions.

### ITCs

#### Acquisition

**Figure 4:**
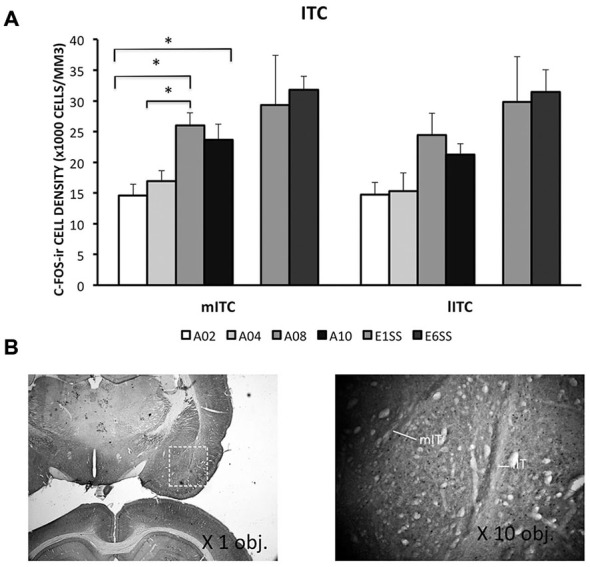
**Densities of c-Fos-ir cells in the lateral and medial intercalated cell cluster (ITC) were depicted**. **(A)** Densities of c-Fos-ir cells in the lITC and mITC. Each data point represents group mean + S.E.M. (*ps < 0.05; *n* = 5–6/group). **(B)** mITC and lITC were defined by GAD67 staining that was visualized by DAB (×1.25 objective lens). C-Fos-ir cells in the mITC and lITC were visualized by DAB-NiCl_2._(×40 objective lens).

ITC area was defined by GAD67 staining as depicted in Figure 4B. The number of c-Fos-ir neurons increased while acquisition proceeded in both medial and lateral ITC (lITC: *F*_(1,33)_ = 11.81; mITC: *F*_(1,32)_ = 8.82; *ps* < 0.05; Table 1). Particularly in the mITC, the number of c-Fos-ir cells was higher on later sessions compared to early sessions, *post hoc* analysis *ps* < 0.05 (Figure 4A; for detailed statistical analysis results, see Table 1). Since ITCs are composed mainly of GABAergic neurons, elevated ITCs activity during acquisition strongly suggests that the inhibitory tone develops as rats are acquiring the avoidance task.

#### Extinction

ITCs activity was greater during the extinction phase than acquisition phase. Although c-Fos-ir cell counts did not differ between ACQ10 and EXT01, a significant increase in c-Fos-ir cell counts was observed in EXT06 in both lITC and mITC, *t*_(10,2.53)_ and *t*_(10,2.44)_, *ps* < 0.05, suggesting transition to extinction did not significantly increase such activity simultaneously, instead, in a delayed mode.

### CeA

#### Acquisition

During the acquisition phase, the number of c-Fos-ir neurons increased with training and peaked in session A08, then reduced to the early acquisition level in session A10. This pattern indicates that CeA may be actively involved in learning active avoidance, but is less involved as learning proceeds to asymptotic performance.

#### Extinction

**Figure 5:**
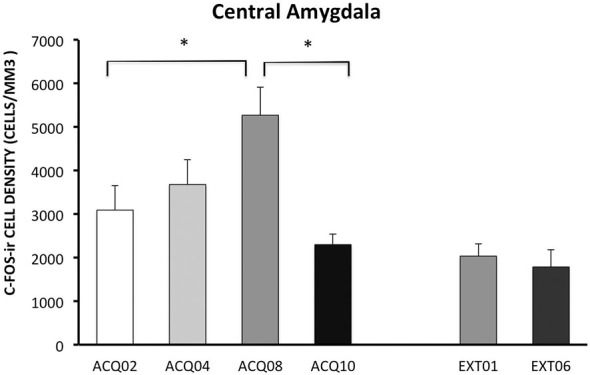
**Density of c-Fos-ir cells in the central amygdala (CeA)**. Each data point represents group mean ± S.E.M. (*ps < 0.05; *n* = 5–6/group).

In contrast to activity in other ROIs, CeA was activated to a lower degree during the extinction phase compared to acquisition phase, *p* < 0.005 (Figure 5). Moreover, CeA neural activity remained at such a low level during the entire extinction phase suggesting that CeA activation may be inhibited when avoidance is acquired and when shock is no longer present (Figure 5, for detailed statistical analysis results, see Table 1).

## Discussion

Here we report differential activity of mPFC and amygdalar sub-regions during lever-press avoidance and extinction. In the mPFC and most amygdalar sub-regions, activity increased in late acquisition sessions (A08–A10) when avoidance response was acquired and peaked in extinction phase when shock was no longer present. GABAergic neurons in the mPFC had a similar pattern, more activated in the mPFC in later acquisition, even more in early extinction (E1), but less activated in late extinction (E6). In contrast, activity in the CeA increased during early acquisition sessions, peaked in A08 and reduced in late acquisition and extinction. Therefore, different patterns of activity were observed in mPFC, BA, LA and ITC compared to CeA. These data suggest that general activity, and particularly inhibitory neuronal activation within mPFC-amygdala circuit shifts in a time-dependent manner during acquisition and extinction of lever press avoidance. Together, these data suggest that altered activity observed in similar regions in the present study using avoidance paradigm in rats and in imaging studies in patients with anxiety disorders (Schwartz and Rauch, 2004; Cottraux, 2005; Guyer et al., 2008).

The role of mPFC and amygdala in avoidance task has been previously studied, however mainly using lesion technique in rodents (Choi et al., 2010; Moscarello and LeDoux, 2013; Beck et al., 2014). Pre-training lesion provides a useful tool to evaluate well defined structure-dependence of a task, (Wan et al., 1994). However, compensatory changes in response to lesions may complicate interpretation. We recently reported that mPFC, striatum and amygdala neural activity assessed by c-Fos and delta-FosB was associated with avoidance and extinction in lever-press avoidance (Jiao et al., 2011; Perrotti et al., 2013). However, these studies only evaluated time points at asymptotic avoidance performance and at the end of extinction learning. In order to understand the role of amygdala and mPFC in the acquisition and extinction processes of avoidance, the present study monitored neural activity at various time points during avoidance acquisition and extinction.

The importance of mPFC neural activity in active avoidance and extinction has been recognized and appreciated in recent works using lever-press or shuttle-box avoidance paradigms (Duncan et al., 1996; Jiao et al., 2011; Moscarello and LeDoux, 2013). These studies demonstrated that avoidance learning induced prominent c-Fos expression in the mPFC and CG (Duncan et al., 1996) while IL lesion impaired avoidance learning (Moscarello and LeDoux, 2013). We reported that rats that failed to extinguish lever-press avoidance exhibited lower c-Fos expression in the mPFC compared to rats that successfully extinguished such response (Jiao et al., 2011). Thus mPFC is actively involved in both acquisition and extinction of active avoidance task in rats.

It is known that mPFC is a heterogeneous structure (Gabbott et al., 1997; Vertes, 2004). In fear conditioning and extinction, PL is associated with fear learning while IL is important in extinction learning (Milad and Quirk, 2002; Quirk et al., 2006; Sierra-Mercado et al., 2011). If fear and avoidance share the same pathway, we would expect greater PL activation during acquisition and greater IL activation during extinction. However, we found that the pattern of c-Fos-ir changes was similar in the PL and IL in avoidance. In support of our data, Moscarello and LeDoux (2013) reported that IL is needed to acquire shuttle avoidance, and to reduce warning signal-elicited freezing, yet PL lesion did not affect acquisition. In shuttle avoidance, the cue that is initially paired with the shock induces fear and facilitates freezing behavior, subsequently preventing a shuttle response. Thus fear needs to be overcome while shuttle avoidance is being acquired. Our results support and extend those of Moscarello and LeDoux in that IL activity is increased during lever press avoidance was acquired. In contrast to Moscarello and LeDoux, PL neural activity was also increased during acquisition of lever press avoidance; these differences may due to different avoidance paradigms used in these studies. We also observed an interesting trend on the activation of PV-ir neurons in this area. While there are more activated PV-ir neurons following A10, there is a trend showing increased number of activated PV-ir neurons after E1 (e.g., CG) and decrement after E6 (e.g., IL). Given c-Fos-ir cell counts remained similar following A10, we speculate that there might be increased excitatory activity in the mPFC during late extinction. However, this speculation requires further investigation.

In the ITCs, c-Fos-ir expression progressively increased as learning proceeded from acquisition to extinction of lever-press avoidance. Accumulated evidence demonstrates that ITCs is critical for fear extinction, specifically, for the expression of extinction (Herry et al., 2008; Likhtik et al., 2008; Mańko et al., 2011). This cell cluster receives input from vmPFC and modulates fear extinction through the CeA, a feed-forward inhibition mechanism of extinction (Quirk and Gehlert, 2003; Milad et al., 2004; Hefner et al., 2008; Likhtik et al., 2008; Mańko et al., 2011). Thus the greater ITCs activity here could lead to reduced “fear” component in late acquisition sessions and in extinction via increasing excitatory input from mPFC neurons. Based on the present data, we speculate that when animals reach near asymptotic avoidance performance (i.e., receiving very few shocks), CeA activity is suppressed by increased ITCs input induced by enhanced mPFC activity.

As described above, we observed an inverse relationship in neural activity between CeA and mPFC-ITCs circuits, which is an increase of c-Fos-ir cells counts in the mPFC and non-CeA amygdala in late acquisition accompanied by a decrease of c-Fos-ir cell counts in the CeA following A08. The inverse relationship of c-Fos-ir in the mPFC and CeA is supported by the anatomical connection between these two structures and their physiological roles in aversive learning that we addressed earlier (Morgan and LeDoux, 1999; Rosenkranz et al., 2003; Amano et al., 2010). Lesion/deactivation in the CeA facilitated shuttle avoidance by reducing freezing (Choi et al., 2010; Moscarello and LeDoux, 2013), suggesting that CeA activity inhibits the acquisition of an active avoidance task. Thus rats exposed to shocks would have high CeA activity during early acquisition phase when avoidance response has not yet been fully acquired. However our study showed that the peak CeA activation occurred on session A08 but not A02 when avoidance responding is near asymptote. Similarly, higher c-Fos-ir in the CeA has been reported in rats that are “good” avoiders compared to “poor” avoiders in shuttle-box avoidance, suggesting that elevated CeA activity is associated with active avoidance learning (Martinez et al., 2013). Other than freezing, CeA is associated with arousal, sympathetic and parasympathetic responses to stimuli (LeDoux, 2007). It is possible that elevated CeA activity is due to other factors such as valence (i.e., bad/good behavioral outcome) state (Moul et al., 2012). In addition, it is known that CeA is highly heterogeneous, for instance, a large portion of CeA neurons are interneurons that inhibit CeA output (McDonald and Augustine, 1993; Pitkanen et al., 1997; Sah et al., 2003). Thus it is possible that the high CeA activity on session A08 could due to increased interneuron activation and lead to reduced CeA output. Therefore, the highest CeA activation observed on A08 may be the result of accumulative neural activation, but not necessarily indicate highest levels of fear.

In addition, our findings indicate that both LA and BA regions remained active during extinction of lever-press avoidance. The involvement of BA and LA in acquisition is expected since this region is necessary to acquire and perform an active avoidance task (Silveira et al., 2001; Anglada-Figueroa and Quirk, 2005). However, the extended activity during extinction suggests that the extinction of active avoidance requires both structures. We also found elevated activity in inhibitory PV-ir cells in the LA during extinction. As BA receives robust inputs from LA, increased inhibitory activity in the LA may lead to decreased output to the BA. Moreover, increased PV-ir neuronal activity was observed in BA following A10 and remained the same during extinction while overall BA activity was higher following E1, suggesting different neuronal population may be involved. For instance, BA neurons that fired to fear-associated CS or extinction-associated CS are innervated by projections from heterogeneous origins such as ventral hippocampus or mPFC (Repa et al., 2001; Herry et al., 2008). Thus, it is possible that activities from distinct neuronal populations associated with acquisition or extinction of avoidance learning are overlapping during late acquisition and early extinction, resulting in different activity patterns in the BA through LA.

It is important to note that the neuronal activity of the brain areas investigated here did not change in relation to shock number. Early in training, rats experienced the most amount of shocks in A01 and then reduce number of shocks through out the acquisition phase. In contrast, mPFC and amygdala sub-regions, except CE, had neuronal activity increasing through out the acquisition phase, and some even through extinction when there were no shocks experienced. Even activity in the CE nucleus did not exactly reflect shock number as its activity was highest at A08, but not A01. Previous studies have implicated that fear is greatest early in avoidance training and gradually reduces as avoidance is learned (Coover et al., 1973; Servatius et al., 2008). Thus, the neuronal activity reported in this study does not exactly correlate with the expected dynamics of fear during avoidance learning. Moreover, previous studies show that activity in the amygdala increase during fear conditioning in humans and animals, paralleling the conditioned response (Quirk et al., 1997; Cheng et al., 2007). Another study reported that c-Fos activity in the mPFC was significantly increased in rats acquiring wheel-turn avoidance compared to yoked and house-exposure control rats (Coco and Weiss, 2005). In addition, increased number of mPFC c-Fos-ir neurons was reported following fear extinction compared to unpaired CS/US control groups (Kim et al., 2010). Thus, these data suggest that the changes in neural activity likely results from the development of behavioral avoidance and extinction, as our observation indicates that there is a lack of association between c-Fos-ir cell counts and shock number received from groups (Figures 1C, 2A, 3A, 4A, 5). Taken together, the results of the present study suggest that activity of mPFC-amygdala during avoidance learning is not merely reflecting fear.

Limitations: The evaluation of c-Fos expression only indicates association of these regions in avoidance acquisition and extinction, but not the necessity of these regions in avoidance learning or extinction. Further investigation is needed to make mechanistic conclusions of these brain regions in avoidance and its extinction. Our results further suggest that to move forward, selective lesions of different cell populations within the mPFC and amygdala is necessary. Future studies should also include other regions such as nucleus accumbens (Ramirez et al., 2015) and striatum, as they are important in motivation and stress controllability.

In conclusion, we demonstrated that the dynamic interaction between mPFC and amygdalar sub-regions could partially be the underlying mechanism of avoidance acquisition and extinction. Thus the network activity in avoidance may *not* be the same in fear conditioning, while there are common structures involved. Our findings on GABAergic neural activity in acquisition and extinction of active avoidance may shed a light on better understanding the mechanism of avoidance and benefit extinction-based therapy for anxiety.

## Conflict of Interest Statement

The authors declare that the research was conducted in the absence of any commercial or financial relationships that could be construed as a potential conflict of interest.
